# “I don’t need my patients’ opinion to withdraw treatment”: patient preferences at the end-of-life and physician attitudes towards advance directives in England and France

**DOI:** 10.1007/s11019-014-9558-9

**Published:** 2014-04-01

**Authors:** Ruth Horn

**Affiliations:** The Ethox Centre, Nuffield Department of Population Health, University of Oxford, Old Road Campus, Rosemary Rue Building, Oxford, OX3 7LF UK

**Keywords:** Decision-making for incompetent patients, Advance directives, Patient preferences at the end-of-life, England, France, Qualitative study, Comparative approach

## Abstract

This paper presents the results of a qualitative interview study exploring English and French physicians’ moral perspectives and attitudes towards end-of-life decisions when patients lack capacity to make decisions for themselves. The paper aims to examine the importance physicians from different contexts accord to patient preferences and to explore the (potential) role of advance directives (ADs) in each context. The interviews focus on (1) problems that emerge when deciding to withdraw/-hold life-sustaining treatment from both conscious and unconscious patients; (2) decision-making procedures and the participation of proxies/relatives; (3) previous experience with ADs and views on their usefulness; and (4) perspectives on ways in which the decision-making processes in question might be improved. The analysis reveals differences in the way patient preferences are taken into consideration and shows how these differences influence the reasons physicians in each country invoke to justify their reluctance to adhering to ADs. Identifying cultural differences that complicate efforts to develop the practical implementation of ADs can help to inform national policies governing ADs and to better adapt them to practice.

## Introduction

Respect for patient autonomy is an essential element of modern medical ethics (Beauchamp and Childress 2008). But there are instances in which a patient is not able to communicate her will, such as when people are kept alive in chronic and at times critical condition. These kinds of cases raise difficult questions about how to respect people who cannot communicate. For several years, advance directives (ADs) have been discussed, first in the United States and then in Europe, as one possible mechanism for enabling a person to communicate, prior to any loss of competence, her will regarding specific treatment refusals.

Several European countries, including England and France have accorded legal status to ADs and the Council of Europe (2009, 2012) has recommended that all member states should adopt such legislation. Yet, the implementation of ADs remains problematic and raises numerous ethical, legal and social questions. One difficult question is: to what extent and under what conditions should an anticipated treatment refusal be respected? I argue that answering this question depends on an understanding of the role patient preferences play in different countries. This understanding gives rise to differences in the problems associated with ADs and their potential role in different cultural contexts.

This paper aims to examine England and France, two countries that value patient autonomy differently. Whereas the situation in England is influenced by a culture that emphasises respect for individual wishes, the French situation reflects a culture promoting the protection of the vulnerable person, even if this is to the detriment of the person’s autonomy. Surprisingly, the implementation of ADs is not significantly stronger in a context that values respect for patient wishes than in a context where patient autonomy is not the overriding principle. This paper will show how the different value accorded to patient preferences influences the reasons physicians in each country invoke to justify their reluctance to adhering to ADs. Identifying cultural differences that complicate efforts to develop the practical implementation of ADs can help to inform national policies governing ADs and to better adapt them to practice.

## Background

In England,^1^ patient autonomy has a central place in health care law, and it underpins the respect that must be granted to a competent patient’s refusal of treatment (*Airedale NHS Trust v. Bland* [1993]; *Re B* (*adult*: *refusal* *of treatment*) [2002]; Foster 2009) whether the reasons for this are rational, irrational or even absent (Re T (*adult: refusal of treatment*) [1993]). Even where a patient has lost competence, a patient’s wish as expressed in an AD has long been considered binding in common law. Since the Mental Capacity Act 2005 fully came into force in 2007, ADs to refuse treatment are a part of the statutory law. As already formulated in the common law (*Re T (adult: refusal of treatment)* [1992]; *Re AK* *(medical treatment: consent)* [2000]; *HE v A Hospital NHS Trust* [2003]; *W* *Healthcare NHS Trust v H and others* [2004]), an AD must be issued voluntarily by a competent and sufficiently informed patient, and apply to the circumstances that have arisen. In a case where an AD concerns the withdrawal of life-sustaining treatment, the Act additionally requires that the directive must be written, signed and witnessed, and clearly states that the decision is to apply it even if life is at risk. Under the Act, the patient can also appoint a “lasting power of attorney”. This clause allows patients to empower someone to make health care decisions on their behalf when they have lost the capacity to decide for themselves.^2^

Since *Airedale NHS Trust v Bland* [1993], English law emphasises that, where an incapacitated patient has made no clear statement when she was still competent, life-sustaining treatment should be withdrawn when it is no longer in the patient’s best interests. When determining the patient’s best interests, the Mental Capacity Act states in part 1.4 that the physician must “so far as reasonably practicable, permit and encourage the person [even with impaired capacity] to participate, or to improve his ability to participate, as fully as possible” in the decision. The physician must also consider “the person’s past and present wishes and feelings”, her “beliefs and values that would be likely to influence his decision if he had capacity”.

In France, the option for patients to write an AD was introduced in 2005 by the law on patients’ rights and on the end of life (Loi no 2005-370). There is no evidence that anticipated treatment refusals were previously recognised in French jurisprudence. As now stipulated in Article L. 1111-11 of the Public Health Code, the doctor can take ADs into account but is not obliged to do so. The patient’s will, as expressed in such a directive, is indicative rather than determinative (Feuillet Le-Mintier 2011). The law states that the doctor alone makes the decision to withdraw life-sustaining treatment. Yet, they are advised to consult a colleague, the patient’s representative, the family, close persons and, if one exists, the AD. Despite its non-binding character, an AD must have been issued less than three years ago. In the absence of a AD, there is no specific requirement to find out what the patient’s wishes would have been. A comparison of European states has found that France confers the weakest power to proxies or surrogates in decision-making (Lautrette et al. 2008).

The same law that introduced ADs extended a competent patient’s previous right to refuse “a” treatment (Loi no 2002-303) to the right to refuse “any” treatment including clinically assisted nutrition and hydration (article L. 1111-4 Public Health Code). As also in the previous law of 2002, the same paragraph stipulates that the doctor has to respect the patient’s wish; yet where the treatment refusal endangers the patient’s life, the doctor should “do all that is possible in order to convince the patient” to continue the treatment. Furthermore, it has been added in the new law of 2005 that “in any case, the patient has to repeat his/her decision after a reasonable lapse of time”, and “the decision must be recorded in the patient dossier”. A patient’s decision to refuse a life-sustaining treatment is considered as so unreasonable that the patient has to repeat their will twice and the doctor must take measures to prevent accusations of negligence. Thouvenin (2011) points out that in a clause that establishes a patient’s subjective right, in other words, that defines the patient as the right-holder, such limitation appears to be a paradox.

In spite of the different legal value accorded to ADs in England and France, the number of written directives is insignificant in both countries (Pennec et al. 2012; Seale 2006a, b; Schiff et al. 2000). Even in countries, such as the United States, where the Patient-Self-Determination Act has since 1990 accorded legal force to ADs, report problems in the uptake of these documents (Hanson and Rodgman 1996). These findings have prompted authors such as Fagerlin and Schneider (2004) to argue that ADs have failed. They interpret the small numbers of ADs to demonstrate that only a few people know what they want, or/and can articulate their wishes, and that many fear misinterpretations of their ADs or do not believe that they would be taken into consideration.

Fagerlin’s and Schneider’s observations concerning the difficulties to articulate future treatment wishes, the fear of possible misinterpretations of ADs, and miscommunications between physicians and patients raise two overarching questions regarding the role of physicians in discussing ADs: (1) How do physicians discuss future treatment options with patients and may they better help them express their wishes? (2) How do physicians take into account patient preferences in decision-making, and what are the arguments and underlying social values for doing (or not doing) so in different legal contexts?

Numerous papers reflect on ADs from a legal or ethical perspective (Buchanan 1988; Michalowski 2005; Feuillet Le-Mintier 2011) and some empirical studies examine the views of patients (Seymour et al. 2004; Rurup et al. 2006). Others, based on questionnaires, focus on physicians’ attitudes towards ADs (Sahm et al. 2005; Rodriguez-Arias et al. 2007; Bond and Lowton 2011). Yet, if we want to better understand why English and French physicians may promote (or not) the writing of such directives, it appears appropriate to take into account, not only their attitudes towards ADs, but also their general attitudes towards patient preferences and how these attitudes reflect legal and practical constraints which echo different cultural traditions (Cartwright et al. 2007; Menaca et al. 2012; Evans et al. 2013). This paper explores problems physicians from different contexts evoke with regard to end-of-life decision-making and shows how the place accorded to patient preferences influences the potential role of ADs in ethics guidance and policies governing ADs in England and France. A better understanding of national differences and of perceived problems rising in a specific context is much needed to inform the development of ADs in European policy-making.

## Methods

This paper presents the results of 28 semi-structured face-to-face interviews on physicians’ views on ADs, and more generally on their experience in making end-of-life decisions for incompetent patients. In 2011, 14 English and 14 French physicians were recruited from university hospitals (n = 2 in England, n = 3 in France) in two different cities in each country. The focus was on doctors working in services which take care of seriously or terminally ill patients (specifically, oncology, nephrology, neurology, geriatrics and palliative care).

Ethical approval for the study was sought in England from an NHS Research Ethics Committee; in France, the Commission Nationale de l’Informatique et des Libertés confirmed that no specific approval procedure was needed for this study. However, access was negotiated with the head of the hospital services and appropriate standards for interviews set out, including guarantees of the anonymity of participants. In England, according to the requirements of the local research ethics committee, physicians were approached after initial contact by the medical director of each hospital, who invited them to contact the researcher if they wished to participate in the study. Each participant received an information sheet about the study and written consent was taken prior to the interviews.

Interviews were conducted by an experienced sociologist in a quiet room in hospitals. Each interview lasted approximately 45 min, was audio recorded and transcribed.

The aim of the interviews was to better understand the importance of a stated treatment preference, and thus to better grasp the potential role of ADs. For this purpose, the interviews examined the following broad themes: (1) problems that emerge when deciding to withdraw/-hold life-sustaining treatment from both conscious and unconscious patients; (2) decision-making procedures and the participation of proxies/relatives; (3) previous experience with ADs and views on their usefulness; (4) perspectives on improving the decision-making processes in question.

The analysis of data gathered for each predefined theme involved numerous readings of the transcribed interviews. This was followed by identifying and refining comparable recurrent themes and patterns that came out during the interviews and that describe English and French physicians’ attitudes and experiences.

Participants are identified according to their nationality (En/Fn), gender (m/f) and their medical specialty (oncology: onc; palliative care: pall; geriatrics: ger; nephrology: neph; neurology: neur; intensive care: intens; rheumatology: rheum; surgery: surg). Without aiming to make generalised claims about “all” English or “all” French physicians, the data echo tendencies which are also apparent in each country’s legislation, public debates, and professional guidelines (Horn 2012).

## Findings

The following findings are based on interviews illustrating the experiences of 14 English and 14 French physicians. Out of the 14 English physicians, 3 were oncologists, 3 neurologists, 3 palliative care specialists, 3 nephrologists, and 2 were geriatrics. In France, out of 14 physicians, 3 were oncologists, 3 neurologists, 3 palliative care specialists, 2 nephrologists, 2 were geriatrics, and 1 doctor worked in intensive care. 7 out of the 14 English physicians and 5 out of the 14 French physicians were women. The small sample size does not allow us to show differences between physicians of different specialties or according to their gender. Cartwright et al. (2007) suggest that such characteristics are rather insignificant compared to national differences, however.

## Respect for patient preferences when deciding to withdraw/-hold life-sustaining treatment

### English physicians’ concerns to know their patients’ wishes

English physicians who were asked about difficulties they encounter when deciding to discontinue treatment for both competent and incompetent patients reported problems regarding patient consent, the timing, and the prognosis. All 14 English doctors interviewed in this study explained the importance of discussing decisions to withdraw/-hold treatment with the patient. The majority of physicians (8/14) considered that these discussions should take place *“when things get worse”* and *“before [bad situations] actually occur”* [E7/f/ger]. Nine doctors evoked their particular concern about *“making a decision on behalf of somebody else”* [E1/m/onc] who is unable to communicate their will and to consent to the decision. Such situations present dilemmas for physicians according a strong value to patient preferences and discussion.

When reflecting on reasons why patient wishes are not always known, most English physicians (8/14) referred to problems regarding the “right” timing to have discussion about future treatments. They explained that often they get to know their patients when they are already *“quite a long way down the road”* and can *“have no meaningful conversation anymore”* [E4/m/ger]. This problem was reported particularly by five doctors working in geriatrics or with chronic neurodegenerative diseases. These physicians considered having only limited experience in discussing future treatment preferences with the patient.

Other physicians (3/14) acknowledged that even if their patients are competent they might delay discussion on future treatment because it means *“acknowledging that life is now limited”* [E1/m/onc]. In his work on prognosis, Christakis (1997) has shown that this problem is inherent in medical profession. Since medical science has improved and doctors are progressively supposed to *“eradicate disease”*, deterioration of the patient’s health condition and death are seen as a failure *“not just of the therapeutic armamentarium to achieve its objective, but also of the physician to fulfil his or her social role”* (Christakis 1997, p. 314).

One doctor in this study added weight to Christakis’ view when she explained how she avoids confronting the patient and herself with a poor prognosis:

In using the lack of information [about possible outcomes] as excuse, we still collude with ourselves to not say how bad it is. Partly because it’s not always bad, and partly because we’ve not found the words; and partly because it’s pushing stones uphill. I think quite a lot of this is just an excuse for not facing our own mortality, rather than a really good reason for not raising the subject. [E3/f/pall]

This physician and her colleagues use the uncertainty about the course of a disease to avoid facing deeper concerns, such as the patient’s and their own mortality.

The interviews suggest that English physicians seem to be torn between their wish to respect patient preferences, which is emphasised in law and professional guidelines (Mental Capacity Act 2005; General Medical Council 2008, 2010; Liverpool Care Pathway 2012) and their unease about communicating a bad prognosis. Hence, deciding to discontinue life-sustaining treatment where the patient’s wish is not clearly known is a dilemma for English physicians.

### French physicians’ difficulties in withdrawing or withholding treatment

The situation is different in France. Following the developments in other European countries, the law of 2005 has been presented by its authors as an important step to introduce greater focus on patient autonomy at the end of life (Sicard 2012). Yet, as Thouvenin points out, the actual goal of the law which was drafted by a commission presided by a physician, Jean Leonetti, was not only to strengthen patient rights at the end of life (Assemblée Nationale 2008b, p. 404–405). By clarifying the conditions under which a physician can withdraw/-hold life-sustaining treatment in accordance with their professional opinion, the law principally focuses on reassuring physicians of the legitimacy of their acts.

Though the law emphasises that medical decisions should be informed by patient preferences it also makes clear that the judgment about the usefulness of a treatment belongs to the physician alone (see article L. 1111-4 Public Health Code; Thouvenin 2011).

Echoing this situation, one of the French doctors interviewed explained:

I don’t need my patients’ opinion to withdraw treatment […] If I think that the patient shouldn’t be resuscitated, that she has no chance, I don’t need her opinion for this. [F11/m/onc]

This physician refers to the law of 2005 allowing the physician to withdraw or withhold treatment according to their judgment and without being obliged to respect the patient’s wish. Whereas some French physicians start discussing the importance of critically evaluating the benefit of treatment at an advanced stage of illness, this has not always been so.

French doctors favoured for many years active treatment even at the end of life and the law of 2005 did not immediately change this well-entrenched attitude. Still today, active interventions continue being of particular value for physicians. (Assemblée Nationale 2008a; Horn 2011)

One French doctor explained that she “never” stops treatment,

…because I’m the one who starts it and many patients actually prefer to die from a chemotherapy, rather than from cancer. […] I don’t want to disappoint my patients. I think by withdrawing treatments we make a moral judgment of the value of the patient’s life, which I don’t want to do at all. […] Recently, I felt like a traitor when the palliative care team persuaded me to stop a treatment although the patient wanted to continue. I was glad when the patient came back to me after a few days in the palliative care service […] I think that patients ask for treatment because this is how our relationship works. [F7/f/onc]

This doctor did not adopt a critical view on medical interventions and gives a strong account of her understanding of the doctor-patient relationship based on active treatment. Treating a patient has moral significance for this physician who compares withdrawing treatment with judging the value of life. Therefore she feels like she is betraying her patients when she stops providing treatment. Later, this doctor explained that while she respects a patient’s wish to continue treatment even when this becomes harmful, it is very difficult for her to respect a treatment refusal.

Although these two doctors express different viewpoints about futile treatment they both share a similar view on patient wishes. Where the doctors have a strong opinion about the medical decision they do not consider the patient’s will. The latter is evoked only in cases where it supports the physician’s decision. The second doctor quoted refers to the patient will in order to justify her decision to continue futile treatment; a decision that is now questionable under the law of 2005 (article L. 1110-5 Civil Code).

Several French doctors (5/14) confirmed that although the law states that physicians must not insist on “unreasonable” treatment, the persisting problem in France is that “most physicians still maintain a curative perspective and always want to go further in order to avoid death” [F3/f/neph]. One doctor even called this attitude the “barbarism of French doctors” [F11/m/onc].

Nevertheless, the law of 2005 seems to have some impact on French physicians, at least on their discourses. The majority of the doctors that have been interviewed (10/14) thought that although it is indeed difficult for them to do so, it would be important to limit interventions at the end-of-life. One of the doctors explained here that:

there are social rules […] and we have to avoid that after three months people end up in a vegetative state. […] That poses the question of how much will this cost the society. And, then we also have to ask what the emotional and social burden is for the family? [F1/m/intens]

The consequences of unreasonable medical interventions are considered from a social perspective, rather than from an individual perspective. Other physicians interviewed also focused on a range of aspects that did not take into account the individual wish. For example, half of the physicians (7/14) listed criteria, such as *“professional consensus”* or *“medical criteria”* (such as increasing somnolence and respiratory insufficiency) or *“families’ preferences”*, according to which they decide to stop or to continue treatment for incompetent patients. Other than their English colleagues, none of the doctors evoked previous patient preferences as a decision criterion.

These interviews revealed, despite changing attitudes, a strong commitment among French physicians to intervene therapeutically to maintain life, and some doctors attribute a moral value to medical interventions. Yet, there is also a growing awareness of the problems entailed by this attitude. The French doctors interviewed perceived these problems from a collective, social perspective, rather than from that of the individual patient.

## Decision-making procedures and the participation of third parties

### English physicians and the duty to empower patients

The English doctors’ focus on patient wishes was also apparent in the way that all 14 English doctors discussed the importance of giving information and helping the patient to make decisions. In this they apparently agreed with the Mental Capacity Act and professional guidelines. They valued absolute respect for patients’ decisions if they *“can be sure that the patient understands the situation”* [E13/m/onc]. One English physician explained that if he has given accurate information and the patient refuses the treatment as a result, he feels

very comfortable with that [because] it’s a sign of a strong, as well as empowered patient, if they feel they can turn around and say, ‘That’s not for me’. I just want to find out whether there is something I could change. [E1/m/onc]

This comment and the following quote show how the certainty about a deliberate patient decision allows these doctors to weigh respect for the patient wish against their duty to safeguard patient welfare:

There is a conflict [of duties] but I’m absolutely sure that a ‘bad decision’—as I would see it—from the patient does not mean that they should be overridden or that they don’t have capacity. […] The issue is…does the patient really understand and was the decision made at a time when they had capacity? [E4/m/ger]

The reflected decision of an informed patient is decisive for these physicians even where a patient decides against their opinion. One doctor explained how she integrates such a decision in her relationship with the patient:

At the end of the day my duty is to care, [that is] to help the patient, even if it doesn’t feel like it’s the right decision that I would make, but that’s their choice. And then my focus will change onto ‘how can we make life better for this person and ensure they are as safe as they can be within what they want to do?’ So, I think I have to accept it. [E7/f/ger]

For this doctor, professional duties are not limited to clinical criteria. She perceives her duty as one of serving the patient according to their individual needs and preferences.

Even in the case where a patient is incompetent, and has no valid AD, most English doctors (10/14) reported that they try to take into account all opinions, such as those expressed in previous statements or by the family, friends, carers, the general practitioner, etc. This attitude echoes the recommendations regarding the best interests assessment as defined in the Mental Capacity Act, stating that physical well-being should be balanced with emotional/psychological well-being by taking into account the incapacitated person’s values, past preferences or present feelings.

Most physicians (8/14) explained that it is important to get the family *“on board”*, to discuss with them and know their view. Yet, all of these physicians explained that they would never entirely rely on the family’s opinion because they do *“not always act in a patient’s best interests”* [E8/f/neph] and according to their wishes. They explained that most families do not really reflect on previous values of the patient but insist on treatment *“partly out of fear that they’ll be seen as the agent of the death of somebody they love”* [E2/m/nep]. Therefore, a physician [E5/f/rheum] told that although she discusses decisions with the family, she makes clear that she alone bears the responsibility for it (see also Kitzinger and Kitzinger 2012).

The priority English physicians set on individual patient wishes points to a libertarian tradition which goes back to authors such as Locke (1993) or Mill (2005), as well as to the Protestant influence backing the right to make one’s own decisions (Dickenson 1999). Yet, physicians are also concerned to balance the right to free choice with their professional duty to ensure welfare interests of a vulnerable patient. It is against this background that the physicians want to rely on an autonomous wish that is on a reflected decision made when the patient was competent and has understood the consequences of the decision. English law also focuses on autonomous decision-making. Yet, in medical practice as well as in jurisprudence (Coggon and Miola 2011), the implementation of autonomy is not always a matter of course. It often seems to remain a theoretical ideal.

### French physicians’ responsibility to protect the patient

Most French doctors (10/14) also emphasised the importance of informing the patient before asking their opinion. Yet, half of these physicians (5/10) believe that they should present their clinical opinion to the patient because *“the final decision remains medical”* [F2/m/ger]. One physician explained that regardless of what the patient wishes *“in the end, we win, the patient loses”* [F1/m/intens]. Another physician, although recognising the importance of respecting the patient’s wish, thought that it is often more important to

reassure the patient, to explain that the staff is responsible for them, that it’s our business to preserve their humanity, protect their dignity and to explain that we are the guarantors of their dignity. […] What is important for us is the social guarantee we can give the patient because we are a country with social values. [F8/m/onc]

The values at stake are humanity, dignity and solidarity. This doctor wants his patients to feel that the medical body acts according to social values and takes responsibility for patients in need.

A similar understanding of the patient as being part of and protected by the society is presented by another French physician who thought that

patients have not only rights, but also duties; the duty to consider the other. I think that our society is progressively a ‘right-to’ society and thus it’s up to the patient to decide. It’s pseudo-individualism and I think that the perversion of this is that the patient should be alone responsible for everything. But the decision is a collective one and is more global. [F14/m/neur]

This doctor refuses the primacy of decisional autonomy and justifies his opinion by a particular view of the individual tied into the society through her duties towards others. The individual is not alone responsible for her decisions because these are “collective” in a broader social sense. For this physician, “collective” decisions do not seem to mean shared decision-making. Rather, he appears to be in line with the French National Ethics Committee (CCNE) which stated in 2000 that medical decisions should be in accordance with the society’s values. More precisely, the Committee considered that the physician is a *“representative of the community”* who *“defends and promotes the values of the society”* (CCNE 2000, p. 11). This perspective alludes to Rousseau’s (1954) understanding of the relationship between the individual and society. According to Rousseau’s idea of the social contract, every individual’s opinions and preferences are subject to the general will which represents the interests of the society as a whole. The general will need to be guided by individuals which are concerned with the public interests and which want to promote social harmony and cooperation. The CCNE attributes this role to physicians. Such an understanding endorses the subordination of individual patient preferences to socially sanctioned medical opinions (see also Horn 2012, 2013; De Vries et al. 2009)

When French doctors reflected on the participation of third parties in the decision-making process, they explained, similarly to the English physicians, that they do not want to rely too heavily on the family. One French physician explained that *“it will be too difficult and violent for [the family], and they might break down”* [F13/m/neur] if they are expected to make decisions about treatment limitations. Like the English doctors, the French physicians want to ease the family’s feelings of guilt. Yet, unlike the English doctors interviewed, none of the French physicians expressed concerns that the family might not be best able to communicate the patient’s values and preferences. On the contrary, two doctors working with amyotrophic lateral sclerosis patients explained that if the family takes a stand (whether positive or negative) on a heavily invasive treatment such as a tracheotomy, they would tend to follow the family’s request, regardless of what a patient may have expressed at an earlier point. In the end, these doctors explained that it is the family who has to care for the patient at their home, and following their wish *“prevents also maltreatment of the patient”* [F13/m/neur].

In the interviews, French doctors expressed their view about the patient as being a part of society. Individual patient preferences are subordinated to collective values aiming to protect the vulnerable person and to guarantee their social integration. These values are defended by the medical body, who are thus allowed to make decisions on behalf of the individual.

## Meaning, sense and use of ADs

Doctors in both countries described having little experience with written directives and that those they had encountered tended to be written up by *“educated middle class people who are neither sick nor old, thus who do not really need an AD”* [E2/m/neph] (see also Cicirelli 1998). English physicians considered however that often patients make verbal advance statements.

### English physicians’ doubts regarding the genuineness of ADs

Consistent with the importance English physicians accord to patient preferences, all doctors appreciated the idea of ADs and considered them to be completely binding “if” they are valid. *“Knowing what [patients] expect from their medical care”* [E14/f/pall], having *“patients’ feelings written down”* [E10/m/neph] was much appreciated by the doctors. Yet, these statements were often followed by “buts…” or “ifs…” and the majority of doctors (8/14) explained that the main problem actually concerns the validity of these documents because:

[…] they have to be very specific which is actually quite difficult to predict, because you can never cover every eventuality. [E4/m/ger]

So, *“how do you know that’s genuine?”* wondered one physician [E5/f/rheum] and another stated that *“the problem is, it’s sort of time*-*bound, you know, it’s only a snapshot of how someone is feeling at one point in their life […] so, how sure can we be that this is the patient’s wish?”* [E6/m/onc]As already stated earlier, most physicians (10/14) considered that in-depth discussion would enable them to better understand the patient’s wishes. One of them thought that

discussions and conversations, particularly if witnessed and shared by relatives, are far more important than a piece of paper. [E2/m/neph]

Yet, four out of fourteen physicians pointed out that it is in fact very difficult to introduce ADs in the discussion with the patient because they could *“imagine that’s quite scary for them”* [E3/f/pall]. One physician specified:

You don’t really want to be discussing death with them when in fact you’re trying to say to them [that the disease will develop slowly]. […] My head tells me yes, yes we should always ask. And the practicalities of it say well, actually…but you don’t…because it’s uncomfortable. [E4/m/ger]

This English physician faces a conflict between the ideal of knowing patient preferences and his reluctance to tackle difficult discussions about end of life.

The concern about the authenticity of wishes expressed in advance echoes the ethical debate about ADs in the English speaking world. Whereas some authors defend the role of ADs in extending patient autonomy (Buchanan and Brock 1990; Dworkin 1993), other authors question the moral authority of previously expressed wishes and whether our (psychological) personal identity remains the same throughout life or develops continually, depending on various external and psychological factors (Parfit 1984; Dresser 1994). Both arguments share the concern for true patient autonomy. Yet, this seems to be a theoretical ideal. In practice the wish to respect patient preferences is met with an obstacle: the difficulty physicians encounter in discussing prognoses and future treatment options.

### French physicians’ scepticism regarding “Anglo-Saxon” principlism

Whereas English physicians evoked problems regarding ADs as means to express a genuine will, French doctors questioned the idea of an AD itself. Half of the physicians (7/14) interviewed believed that ADs do not have a place in French practice. One doctor pointed out that:

ADs are Anglo-Saxon inventions; it’s typical for them [the “Anglo-Saxons”] to determine and respect all these principles. […] I draw a line between something that is Anglo-Saxon and something that is Latin because I want to give sense to a relationship […] and do not want to resolve problems by signing a paper. [F3/f/neph]

Another French physician explained that *“Anglo*-*Saxon principlism”* leads to the use of all kinds of *“protocols, ADs, do*-*not*-*resuscitate*-*orders, end*-*of*-*life protocols,* etc*.”* without however helping doctors to make decisions. [FP1] The arguments of these two doctors refer to the principlist approach in bioethics (Beauchamp and Childress 2008) which, as Callahan points out, *“reflects the liberal, individualist culture from which it emerged”* (Callahan 2003, p. 288); this, as several doctors noticed, does not fit with the idea that the physician makes socially sanctioned decisions in caring for her patient.

When explaining that it is important to *“remain community*-*minded and make collective, rather than individual decisions”*, one French doctor even considered that *“it’s not ethical to rely only on the patient […]; it’s ridiculous!”* [F1/m/intens]

A minority of physicians (5/14) recognised, however, that although *“the idea [of ADs] comes from the Anglo*-*Saxon countries”*, such directives should be accepted also in France. Yet, they considered that *“on a cultural level French people aren’t there yet”* [F5/f/pall].

And indeed another physician explained that although

the law of 2005 is a real reform and reorientation of medical practices these changes are new and […] the French National Medical Council only recently revised its paternalistic tradition. Prior to this, we learned not to embarrass patients with their illness but treated them in a way that we thought would be good for them. [F6/f/pall]

Thus, before implementing ADs in France,

it would be more important to accept the idea of palliative care, that is, of stopping treatment that has no benefit and to accept that this is not the end [of what physicians can do for a patient]—we have to change our technical, body centred thinking and learn to take into account the benefit, the comfort and the patient’s wish. [F2/m/ger]

It appears that French doctors, who agreed that ADs could be beneficial, referred to a particular meaning ADs have in France. This was pointed out by the author of the law of 2005, Jean Leonetti. He explained that the principal reason for introducing ADs was to *“ease doctors’ feelings of guilt”* when withdrawing life-sustaining treatment (Assemblée Nationale 2008b, p. 237). Patient preferences appear to be secondary. The need to ease feelings of guilt shows how difficult it is for French doctors not to employ all means to cure a patient.

The interviews revealed a long tradition of what is called “acharnement thérapeutique” or “obstination thérapeutique déraisonnable” in the French debate and which could best be translated by “therapeutic determination or relentlessness” and “unreasonable therapeutic stubbornness” (Horn 2011). These terms go beyond what is called in the English-speaking literature “futile treatment” and reveal the strong attachment of French physicians to the value of saving lives at any price. This value is also emphasised by the Catholic tradition whose impact on modern France cannot be denied (Willaime 1996).

## Perspectives on improving the practicability of ADs

### English physicians and practical solutions to improve the use of ADs

Although English doctors questioned whether ADs always express the genuine will of a person, they did nevertheless propose practical solutions for promoting and improving the use of ADs. One doctor suggested that *“proper documentation would probably be useful”* [E4/m/ger]. Others thought that *“everyone [should] carry these smart cards or microchips”* [E5/f/rheum] so that ADs would be available *“in an electronic patient record system”* [E12/f/pall].^3^ Another solution two English physicians [E7/f/ger; E9/m/onc] proposed was to include the general practitioner because they follow the patient from the diagnosis until her hospitalisation.

Doctors supported also the idea of introducing standard forms of ADs in the patient’s medical file:

One thing that would be really, really helpful is if we would have a page on treatment aims in our clerking booklet that we go through, and you have to write the patient’s history and their medication…I think that just advanced thinking about what their ceiling of care is going to be, would actually be really helpful. Just to have that more in the culture of thinking right at the start of coming in. [E5/f/rheum]

Another doctor objected that *“making it bureaucratic, distancing it from the personal [aspects] makes it easier for the professional really, but it doesn’t make it that much easier for the patient”*. Therefore, he suggested that formalising ADs has to go hand in hand with more discussion and *“a greater acceptance that [advance*-*care*-*planning] is a normal part of what [doctors] do.”* [E4/m/ger]

The English physicians interviewed allude to the Liverpool Care Pathway, emphasising besides ADs, broader advance-care-planning and doctor-patient communication on end-of-life care. Advance-care-planning can help identifying patients’ general and specific preferences. It thus helps assessing the authenticity of a wish (Horn and ter Meulen 2014) which is important for English doctors when facing the conflict between respect for patient preferences and their concern for patient welfare. Yet, like ADs, advance-care-planning requires facing and communicating bad prognosis which, in practice, makes physicians reluctant to implement either of these possibilities.

### French physicians and their perspectives on improving end-of-life care in general

The majority of French doctors (8/14) also thought that the use of ADs should be improved, but none of them discussed concrete solutions. Instead, their suggestions concerned physicians’ attitudes in general. According to one of them, physicians in France should *“first learn to accept that medicine is not almighty and cannot improve every condition”*. [F11/m/onc]

In order to do so, one doctor would wish to

integrate more of what you call ‘soft sciences’ in France; I mean a bit more of social and human sciences and less science-sciences in our studies as well as amongst our staff members. [F3/f/neph]

This doctor explained further that physicians should not only focus on physiology but should *“know what it means to be empathetic, what emotions are, and which values are important”.* She did not specify which values are important to her. She explained that being a nephrologist she *“has no culture to refuse [treatment]”* because *“discontinuing [treatment] in nephrology is discontinuing a personal relationship [she has] created over many years”.* This physician expresses a strong emotional attachment to her patients. There may be difficulty in withdrawing treatment if doctors cannot adopt the *“detached concern”* that Fox and Lief (1963) observed amongst physicians who learn to be empathetic at the same time as keeping an emotional distance from the patient.

Most French physicians (10/14) believed that they had to learn to question the benefit and aims of treatments before the *“Anglo*-*Saxon”* idea of ADs could be realised [F13/m/neur]. Because their arguments focused on improving their evaluation of what treatment is beneficial in a singular case, the doctors did not mention patient preferences as a criterion to determine the benefit. Even where French physicians adopt a critical view of their practices, patient wishes are not decisive in end-of-life decision-making. In such an environment, ADs are not needed in order to enhance patient autonomy, but remain an exotic idea, difficult to integrate in medical practice.

## Conclusion

Drawing upon an analysis of interviews with French and English doctors, this study shows how patient preferences are taken into consideration in different social and cultural contexts and how these differences influence physicians’ perspectives and attitudes towards ADs. Such an investigation demonstrates context specific reasons for the resistance towards the implementation of ADs, irrespective of whether the cultural and legal context favours respect for patient autonomy—and therefore ADs—or not.

In England, a country with a strong libertarian tradition that values respect for individual wishes and beliefs, physicians consider ADs as important means to enhance patient autonomy. Yet at the same time, the understanding of autonomy as an ideal according to which a person’s wish is authentic and free of influences explains English physicians’ reluctance to the implementation of ADs. Although they believe that assurance about the authenticity of autonomous decisions can be established through doctor-patient discussion, they describe their reluctance to have such communication in the light of bad prognosis and death. In order to better understand the physicians’ difficulties in implementing values that are imbedded in their culture and law, it will be important to further investigate their practices. Such investigation will help to address problems related to the implementation of ADs within a context where patient preferences are valued.

By contrast, countries, such as France, with a rather holistic approach focus on collective, social aspects rather than individual preferences. From such a perspective, the vulnerable person must be protected by the medical body representing and defending universal social values. Therefore decisions can be made on behalf of the patient and according to the professional opinion relaying social values. In such a context, ADs, which imply disclosure, communication, and respect for individual wishes, are perceived as a foreign concept that does not match with the respective medical attitudes (see also Menaca et al. 2012; Evans et al. 2013). Yet, as the example of France shows, things begin to change and it will be interesting to further observe if, and if so, how physicians integrate gradually the new emphasis on patient autonomy in their practice. Further research should also explore whether ADs can be useful in the French context or whether other forms of advance care planning focusing not only on patient wishes but also on physicians’ responsibilities to advise vulnerable patients, could be more effective.

Physicians’ reservations about the practical implementation of ADs vary depending of the role patient preferences play in a certain country. Surprisingly, problems associated with the use of ADs are not lesser in a context that emphasises respect for patient autonomy than in a context that does not see/perceive patient autonomy as the overriding principle. The problems perceived in each context are different and need to be addressed in a specific way. If policy-makers want to improve the implementation of ADs, measures should be taken to reassure English physicians about the authenticity of the patient will. In France, doctors should be reassured that taking into account patients’ wishes does not put in question their professional competency. In both countries, such measures could aim to enhance the recognition of “the relational aspects of autonomy and central human needs for support and communication” (Krones and Bastami 2014, p. 195). Understanding ADs as means to open a dynamic dialogue between physicians and patients could ease tensions that emerged in each country: tensions between physicians’ wishes to respect patient preferences and the difficulty to initiate discussions about such preferences, and tensions between the value of therapeutic interventions and the emerging focus on patient participation.

The study suggests the importance of taking into account different cultural and social contexts in the ethical analysis of ADs as well as in policy-making. Understanding the differences between the physician-patient relationship and the role of patient preferences in England and France helps to inform the kind of policy and ethical guidance that should be developed. It follows that a comparative approach of these countries is an important first step in developing comprehensive recommendations for the use and implementation of ADs.

## Footnotes

1 Although the English legal system essentially comprises England and Wales, this paper focuses on England since this is where the interviews were conducted.

2 This paper focuses only on ADs and will not analyse the situation with regard to proxy decision-making, but for such discussion see Samanta (2009).

3 To date neither England nor France supports a system that would formalise the use of ADs in daily practice.
